# Mr. E. Amis

**Published:** 1889-12

**Authors:** 


MR. E. R. AMIS, Agent for the New York Pharmical Association, was in the City recently, advertising among the profession the elegant and efficacious preparations of Lactopeptine, which seem destined to fill a long felt want.

